# 

**Published:** 1833-10-01

**Authors:** 


### CONTENTS

OF THE

### MEDICO-CHIRURGICAL REVIEW,

No. XXXVIII. OCTOBER 1, 1833.

### REVIEWS.

I.

Observations on Injuries and Diseases of the Rectum. By HERBERT MAYO, Esq.. 289

1. On Laceration of the Rectum.. .. .. .. .. .. .. .. .. .. 289

2. On Protrusion of the Rectum .. .. .. .. .. .. .. .. .. .. 292

3. Bleeding and Pain of the Rectum .. .. .. .. .. .. .. .. .. 294

4. Stricture of the Rectum .. .. .. .. .. .. .. .. .. .. .. 299

5. Cancer Recti .. .. .. .. .. .. .. .. .. .. .. .. .. 303

II.

Outlines of Pathology. By W. P. ALISON, M.D... .. .. .. .. .. .. .. .. 306

III.

The Infirmities of Genius, illustrated by the Habits and Constitutions of Men of Literature and Genius. By R. R. MADDEN, Esq. .. .. .. .. .. .. .. .. 316

III. (bis.)

MORBID ANATOMY.

I. Anatomie Pathologique du Corps Humain. Par J. CRUVEILHIER (17th Livraison) .. .. .. .. .. .. .. .. .. .. .. .. .. .. .. ..

II. Principles and Illustrations of Morbid Anatomy. By J. HOPE, M.D. Part VI. } 329

1. Puerperal Inflammation of Muscles and Membranes .. .. .. .. .. .. 330
2. Hyperæmia of Alimentary Canal .. .. .. .. .. .. .. .. .. .. 332
3. Softening of the Alimentary Canal .. .. .. .. .. .. .. .. .. 336

IV.

The Analysis of Inorganic Bodies. By Professor BERZELIUS. Translated by Mr. REES .. .. .. .. .. .. .. .. .. .. .. .. .. .. .. .. .. .. 337

V.

Researches from the Case-book, to illustrate the Influence of the Mind on the Body, &c. &c. By R. FLETCHER, Esq. .. .. .. .. .. .. .. .. .. .. 341

VI.

Observations on the Illusions of the Insane, and on the Medico-legal Question of their Confinement. From the French of M. ESQUIROL .. .. .. .. .. .. 352

VII.

Transactions of the Provincial Medical and Surgical Association, Vol. I. .. .. 358

1. Theory of the Frontal Sinus—(Dr. MILLIGAN) .. .. .. .. .. .. .. 359
2. Observations on the Peculiarities of the Diseases of Infants—(Dr. MILLIGAN) .. .. .. .. .. .. .. .. .. .. .. .. .. .. .. .. 361
3. Cases illustrating Diseases of the Heart. By Dr. JEFFREYS .. .. .. 365
4. Observations on the different Signs which distinguish the Sac in strangulated Hernia, with practical Remarks. By Mr. JAMES .. .. .. 368

VIII.

A Treatise on the Venereal Disease and its Varieties. By WILLIAM WALLACE, Esq. 372

1. Primary Syphilis .. .. .. .. .. .. .. .. .. .. .. .. .. .. 374

2. Phagedænic Primary Syphilis.. .. .. .. .. .. .. .. .. .. .. 395

3. Phagedænic Primary Syphilis without Slough .. .. .. .. .. .. 397

4. Phagedænic Syphilis, with White Slough .. .. .. .. .. .. .. 405

5. Phagedænic Syphilis, with Black Slough .. .. .. .. .. .. .. 410

IX.

A Treatise the Diseases of the Liver and on Bilious Complaints. By GEORGE H. BELL, Esq. .. .. .. .. .. .. .. .. .. .. .. .. .. .. .. .. 415

1. Sero-hepatitis .. .. .. .. .. .. .. .. .. .. .. .. .. 416

2. Puro-hepatitis .. .. .. .. .. .. .. .. .. .. .. .. .. 417

X.

Theoretisch-Pratisches Handbuch, &c. *i. e.* a Manual of the Theory and Practice of Midwifery. By Dr. FRORIEP, of Weimar .. .. .. .. .. .. .. .. 418

XI.

Considerations Practiques sur les Neuralgies de la Face, *i. e.* Practical Observations on Neuralgia of the Face. By M. HALLIDAY, M.D. .. .. .. .. .. .. .. 421

XII.

The Homœopathic Medical Doctrine, or Organon of the Healing Art; a new System of Physic, from the German of Hahnemann. By Messrs. DEVRIENT and STRATTEN .. .. .. .. .. .. .. .. .. .. .. .. .. .. .. 429

### PERISCOPE.

PART I.

### Spirit of English Periodical Literature.

1. Our Art in Turkey .. .. .. .. .. .. .. .. .. .. .. .. 433
   Physicians in Turkey .. .. .. .. .. .. .. .. .. .. .. 433
   Fees in Turkey .. .. .. .. .. .. .. .. .. .. .. .. 436
   Apothecaries in Turkey .. .. .. .. .. .. .. .. .. .. 438
   Poisoning in Turkey .. .. .. .. .. .. .. .. .. .. .. 438
2. Dr. Graves on the Treatment of various Diseases .. .. .. .. .. .. 439
   Lord Bacon's Remarks on the Sins of Physicians .. .. .. .. .. .. 439
   Fracture of Ribs .. .. .. .. .. .. .. .. .. .. .. .. 441
   Suffocative Catarrh .. .. .. .. .. .. .. .. .. .. .. 441
   Mortification of the Liver .. .. .. .. .. .. .. .. .. .. 443
   Purpura Hæmorrhagica .. .. .. .. .. .. .. .. .. .. 444
   Emmenagogues—Pulse in Hydrocephalus .. .. .. .. .. .. .. 445
3. Danger of examining Females, Dr. Baird's Case—Persecution in Liverpool .. 446
4. Dr. Elliotson on Yellow Vision in Jaundice .. .. .. .. .. .. .. 447
5. Diseases of the Eye in and after Cholera .. .. .. .. .. .. .. 447
6. Croton Oil as a Counter-Irritant .. .. .. .. .. .. .. .. .. 448
7. Carotid Aneurism, by Operation on the Distal Side of the Tumour .. .. .. 448
8. What Syphilis and Mercury can do.. .. .. .. .. .. .. .. .. 449
9. Severe Case of Hydrocephalus terminating in Recovery (Dr. Graves) .. .. 451
10. Umbilical Hernia—Protrusion of Liver .. .. .. .. .. .. .. .. 453
11. Mr. Flemming on Affections of the Septum of the Nose .. .. .. .. 454
    A. Bloody Tumour .. .. .. .. .. .. .. .. .. .. .. .. 454
    B. Abscess of Septum .. .. .. .. .. .. .. .. .. .. .. 454
12. Dr. Ayre on Cholera—(Review) .. .. .. .. .. .. .. .. .. 457
13. Mr. Carmichael on Inflammatory Affections of the Brain and Membranes, with numerous Cases—(Review) .. .. .. .. .. .. .. .. .. .. .. 459

14. Dr. Davis's Principles and Practice of Obstetric Medicine—(Review) Nymphomania .. .. .. .. .. .. .. .. .. .. .. .. .. 465
15. On some Points connected with the Anatomy and Surgery of Inguinal and Femoral Hernia. By G. J. Guthrie, Esq.—(Review) .. .. .. .. .. .. .. 470
16. On the Character of the Esquimaux, phrenologically considered .. .. .. 474
17. Mr. Eastcott on Fatty Discharges from the Bowels .. .. .. .. .. .. .. .. 477
18. Dr. Ashburner and Mr. Belinaye on Sarsaparilla and the Smilax Aspera.. .. 477
19. Mr. Bloxham on Dislocation of the Head of the Femur on the Os Pubis, with Fracture of the Femur .. .. .. .. .. .. .. .. .. .. .. .. .. 480

### PART II.

### Spirit of the Foreign Periodicals.

1. Dr, Otto on Small-pox, Vaccination, &c. .. .. .. .. .. .. .. .. .. .. 481
2. Dr. Hufeland on the Influenza at Berlin .. .. .. .. .. .. .. .. .. .. 484
3. Coincidence of Human Epidemics with those among Fishes. By M. Alibert.. 485
4. Messrs. Chomel, Recamier, Dance, and others, on large Doses of Antimony in Phlebitis and Pulmonary Hæmorrhage .. .. .. .. .. .. .. .. .. 486
5. Clinical Report from La Pitié on Variola and Varioloid .. .. .. .. .. 487
6. Paraplegia in a young Girl, cured by Strichnine.. .. .. .. .. .. .. .. 487
7. Clot Bey on the frequency of Calculous Disorders in Egypt .. .. .. .. 488
8. M. Labat on the Taliacotion Operation in Egypt.. .. .. .. .. .. .. .. 489
9. Gastrotomy in a Case of Extra-Uterine Pregnancy .. .. .. .. .. .. .. 491
10. Professor Lobstein on Vaccination .. .. .. .. .. .. .. .. .. .. .. .. 491
11. Etiology and Treatment of Gangrena Senilis. By M. Dupuytren .. .. .. 492
12. Pommade to preserve the Hair .. .. .. .. .. .. .. .. .. .. .. .. 493
13. Extracts from German Literature.
    1. A wonderful Heart .. .. .. .. .. .. .. .. .. .. .. .. .. .. .. 493
    2. Excision of Articulations.. .. .. .. .. .. .. .. .. .. .. .. .. 494
    3. Hypertrophy of the Mamma .. .. .. .. .. .. .. .. .. .. .. .. .. 494
14. Adhesion of the Placenta .. .. .. .. .. .. .. .. .. .. .. .. .. .. 498
15. Gonorrhœa communicated by swallowing the Discharge .. .. .. .. .. .. 498
16. On the different kinds of Goitre .. .. .. .. .. .. .. .. .. .. .. .. 498
17. Anencephalous Monstrosity .. .. .. .. .. .. .. .. .. .. .. .. .. .. 499
18. Clinical Observations on—
    A. Croup.. .. .. .. .. .. .. .. .. .. .. .. .. .. .. .. .. .. 501
    B. Excision of the Clavicle .. .. .. .. .. .. .. .. .. .. .. .. .. 501
    C. Morbus Coxarius .. .. .. .. .. .. .. .. .. .. .. .. .. .. .. 501
19. Rhinoplastic Operation .. .. .. .. .. .. .. .. .. .. .. .. .. .. .. 502
20. Chemistry of the Serum of the Blood .. .. .. .. .. .. .. .. .. .. .. 502
21. Medical Galvanism .. .. .. .. .. .. .. .. .. .. .. .. .. .. .. .. 503
22. Lithotrity .. .. .. .. .. .. .. .. .. .. .. .. .. .. .. .. .. .. 503
23. Triple Ureter .. .. .. .. .. .. .. .. .. .. .. .. .. .. .. .. .. 504
24. Poisoning by Fumes of Arsenic .. .. .. .. .. .. .. .. .. .. .. .. .. 504
25. M. Bouillaud on Apoplexy .. .. .. .. .. .. .. .. .. .. .. .. .. .. 504
26. Somacetics in relation to Orthopody .. .. .. .. .. .. .. .. .. .. .. 504
27. Exstrophy of the Bladder.. .. .. .. .. .. .. .. .. .. .. .. .. .. 505
28. Inoculation of Syphilis .. .. .. .. .. .. .. .. .. .. .. .. .. .. 506
29. Open Foramen Ovale in an Adult .. .. .. .. .. .. .. .. .. .. .. .. 506
30. M. Portal's Prize-Legacy .. .. .. .. .. .. .. .. .. .. .. .. .. .. 506

31. New Article of Food .. .. .. .. .. .. .. .. .. .. .. .. .. .. .. .. .. .. 506
32. Extra-Uterine Pregnancy.. .. .. .. .. .. .. .. .. .. .. .. .. .. .. .. .. 506
33. On Pellagra.. .. .. .. .. .. .. .. .. .. .. .. .. .. .. .. .. .. .. .. 507
34. Petechial Fever in Italy .. .. .. .. .. .. .. .. .. .. .. .. .. .. .. .. 508
35. Tonospasmia—a new Nervous Disease .. .. .. .. .. .. .. .. .. .. .. .. 509
36. Encysted Abscess of the Cerebellum .. .. .. .. .. .. .. .. .. .. .. .. 509
37. Review of Cruveilhier.
A. Atrophy of the Convolutions of the Brain .. .. .. .. .. .. .. .. .. .. 510
B. On some Diseases of the Muscles .. .. .. .. .. .. .. .. .. .. .. .. .. 512
C. Case of Diaphragmatic Hernia .. .. .. .. .. .. .. .. .. .. .. .. .. 513
38. Dr. Froriep on the Medico-political Regulations against the Cholera—(Review) 513
39. On the Position of the Embryo and Fœtus in the different Classes of Animals. By M. Dubois.—(Review.) .. .. .. .. .. .. .. .. .. .. .. .. .. .. 515
40. M. Chosy on Polypous Concretions in the Heart .. .. .. .. .. .. .. .. .. 518
41. M. Bouillaud on the Sounds of the Heart and Arteries .. .. .. .. .. .. .. 519
42. On Ulcers, Indurations, &c. of the Os Tincæ. By M. Duges and Mad. Boivin, with numerous Cases—(Review) .. .. .. .. .. .. .. .. .. .. .. .. .. 522
43. M. Maingault on Croup .. .. .. .. .. .. .. .. .. .. .. .. .. .. .. .. .. 526
44. M. Mott's Improvements in Medical Electricity .. .. .. .. .. .. .. .. .. 526
45. Dr. Coste on Embryology.. .. .. .. .. .. .. .. .. .. .. .. .. .. .. .. 527
46. Helminthology .. .. .. .. .. .. .. .. .. .. .. .. .. .. .. .. .. .. .. 527
49. Traumatic Tetanus—Cholera—Diptherite .. .. .. .. .. .. .. .. .. .. .. 528

PART III.

### Clinical Review and Hospital Reports.

1. Review of Dr. Macfarlane's Clinical Reports on Burns and Scalds, with numerous Cases, Observations, and Dissections .. .. .. .. .. .. .. .. .. .. 529
2. Dr. Roots' Clinical Lectures—Fever—Porrigo—Acute Rheumatism—Hepatic Abscess .. .. .. .. .. .. .. .. .. .. .. .. .. .. .. .. .. .. .. .. .. 538
3. Clinical Lectures of M. Dupuytren on Fissure of the Anus.—(Review of the Third Volume) .. .. .. .. .. .. .. .. .. .. .. .. .. .. .. .. .. .. .. 542
4. Dr. Latham's Clinical Lectures on the Duration of Fever—(Bartholomew's).. 544
5. Cases of Laryngotomy. By Mr. Arnott—(Middlesex Hospital) .. .. .. .. .. 546
6. Clinical Report of Cases, illustrating the Treatment of Gonorrhœa. By Mr. H. J. Johnson, House Surgeon of the Lock Hospital .. .. .. .. .. .. .. 549
A. Abscess of the Cellular Membrane of the Penis .. .. .. .. .. .. .. .. 559
B. Induration of the Corpus Spongiosum Urethræ .. .. .. .. .. .. .. .. 561
C. Enlargement of the Lacunar Glands of the Urethra .. .. .. .. .. .. .. 561

### Miscellanies.

I. Aldersgate Dispensary .. .. .. .. .. .. .. .. .. .. .. .. .. .. .. .. .. 564
II. Royal College of Surgeons .. .. .. .. .. .. .. .. .. .. .. .. .. .. .. 565
III. Medical Reform—College of Physicians .. .. .. .. .. .. .. .. .. .. .. 566

OBITUARY.

1. Dr. Darwall .. .. .. .. .. .. .. .. .. .. .. .. .. .. .. .. .. .. .. .. 569

2. Mr. Alcock .. .. .. .. .. .. .. .. .. .. .. .. .. .. .. .. .. .. .. .. 571

Bibliographical Record .. .. .. .. .. .. .. .. .. .. .. .. .. .. .. .. .. 571

Extra-Limites.

Mr. Phillips' Letter to the Editor of the Med.-Chir. Rev. .. .. .. .. .. .. .. 573

